# Investigation of Bacteriological Quality of Meat from Abattoir and Butcher Shops in Bishoftu, Central Ethiopia

**DOI:** 10.1155/2019/6416803

**Published:** 2019-05-02

**Authors:** Abebe Bersisa, Dereje Tulu, Chaluma Negera

**Affiliations:** ^1^Southwest Shoa Zone Livestock Development and Fishery Office, Woliso, Ethiopia; ^2^Ethiopian Institute of Agricultural Research, Tepi Agricultural Research Center, P.O. Box 34, Tepi, Ethiopia

## Abstract

The study was conducted from November 2015 to November 2016 to determine bacterial load and identify pathogenic bacteria (*S. aureus*, *E. coli*, and *Salmonellae* species) in meat from abattoir and butcher shops as well as to assess associated hygienic and sanitation practices being experienced in the selected study site. A cross-sectional study was conducted where a simple random sampling method was used to select butcher shops, and the municipal abattoir was purposively selected. A structured questionnaire survey was also used to assess hygienic status of the municipal abattoir and butcher shops. A total of 124 samples (48 swab samples from abattoir carcass, 4 samples of carcass washing water about 20 ml of each, and 36 swab samples each from butcher shop cutting table and cutting knife, respectively) were collected during the study period. The collected samples were processed for aerobic plate count, and the total mean count was found to be 4.53 log_10_ cfu/cm^2^ from abattoir carcass swab samples, 2.4 log_10_ cfu/ml from water samples, 6.58 log_10_ cfu/cm^2^ from butcher shops cutting table, and 6.1 log_10_ cfu/cm^2^ from cutting knife swab samples. *E. coli* was the dominant bacterial species isolated (35.2%), followed by *S. aureus* (22.5%) and *Salmonellae* species (9.9%). According to the questionnaire survey, 48.4% (15/31) of the abattoir workers did not receive any training regarding food safety issues. Moreover, a majority (66.67%) of the respondents of the butcher house workers were grade 1–4 (elementary) in their educational level and do not use hairnet and handle money with bare hands during serving meat to consumers. The study showed that the hygienic status of the abattoir and butcher shops in the study area is poor, and the obtained results of bacterial load are higher than the acceptable limit of the standard. Therefore, the necessary strategies towards hygiene and sanitation of meat in the town should be implemented.

## 1. Introduction

Meat is one of the most perishable foods, and its composition is ideal for the growth of a wide range of spoilage and pathogenic bacteria [1]. It is prone to contamination at various stages from primary production to when it is ready for consumption (farm-to-fork). Contaminated meat is one of the main sources of food-borne illnesses and death caused by agents that enter the body through ingestion [2]. Food-borne diseases are diseases resulting from ingestion of bacteria, toxins, and cells produced by microorganisms present in food [3].

It is generally recognized that the most significant food-borne hazards from fresh meat are bacteria that can cause disease in humans (pathogenic bacteria), such as *Salmonellae* species, *Staphylococcus aureus, Listeria monocytogenes, Campylobacter* species, and *Escherichia coli* O157: H7. Some of these, particularly *E. coli* O157: H7, require only a few bacteria to cause food poisoning in humans. The main sources of contamination are the slaughtered animals themselves, the workers and working environment, and to a lesser degree, contamination from air via aerosols and from carcass dressing water [1, 4]. Moreover, the contaminating organisms are derived mainly from the hide of the animals and comprise organisms that originate from stomachs and intestines, which are excreted in their feces [5].

Meat contamination in abattoirs and retail meat outlets result from the use of contaminated water, unhygienic practices like poor handling, use of contaminated tables to display meat intended for sale, and the use of contaminated knives and other equipment in cutting operations [6]. Knives, wooden boards, and weighing scales from retail shops are sources of bacterial contamination, particularly *Staphylococcus aureus* and *Shigella* species [7].

Testing against microbiological criteria provides a way of measuring how well the operator has controlled the slaughter, dressing, and production processes to minimize and control contamination [8]. Bacterial counts of meat are used as an acceptable indicator of its hygienic quality. The poor infrastructural facilities in slaughter houses, unhygienic animals, and poor handling of carcasses attribute to the high bacterial load in meat. Thus, by assessing the bacterial counts, the threat posed to human health can be ascertained [1]. Food-borne diseases occur commonly in developing countries because of the prevailing poor food handling and sanitation practices, inadequate food safety laws, weak regulatory systems, lack of financial resources to invest in safer equipment, and lack of education for food handlers [9].

Hygienic and quality control methods of meat and meat products, especially in food catering, have been recommended in many countries [10]. Without proper hygienic control, the environment in abattoir and butcher's area can act as important sources of bacterial contamination [11].

The demand and consumption of animal products such as meat (especially raw meat) is high in Bishoftu town in particular and in the country in general. Moreover, Bishoftu is known by natural gifts of lakes that attract tourists and are frequently visited by national and international guests. Thus, understanding the existing situation on food-borne infections and designing appropriate control strategies are mandatory. Nevertheless, reports on the hygienic status and handling practices of meat in abattoir and butcher shops are fragmented because no comparable data are available regarding the assessment of food safety practice, food-borne diseases, and microbial load of meat in the abattoir and butcher shops of the study area. These factors could hinder government's ability to accurately apply measures on the impact of food contamination problems on public health. Therefore, the objectives of this study were to determine bacterial load and identification of pathogenic bacteria (*S. aureus*, *E. coli*, and *Salmonellae* species) in meat from abattoir and butcher shops as well as to assess associated hygienic and sanitation practices being experienced in Bishoftu, central part of Ethiopia.

## 2. Materials and Methods

### 2.1. Study Area Description

Bishoftu is located in central Ethiopia, at a distance of 47 km of the South East of Addis Ababa, the capital city of the country. The town has seven lakes located in different parts that present an excellent opportunity for the development of resorts that contributes to the enjoyable climate and adds color to the town. The richness of the landscape, variety of flowers, and blueness of the lakes attract tourists and are more frequently visited by guests from different parts of the world throughout the year. Bishoftu has a total residential population of 200,000 people, which is rapidly growing [12]. The town lies between 8°35′ N latitude and 39°06′ E longitude and an altitude of 1860 meter above the sea level, and the area has annual rainfall of 871 mm (long rainy season from June to September and short rainy season from March to May and the dry season from October to February). The mean annual maximum and minimum temperatures are 26°C and 14°C, respectively, with a minimum relative humidity of 63.8% [13].

### 2.2. Study Design and Target Groups

A cross-sectional study design was employed, whereby a simple random sampling of butcher shop was carried out and the municipal abattoir which was the source of meat to the butcher houses of the city was purposively selected. Only cattle were slaughtered at the municipal abattoir. Meat surface swab samples, water from abattoir, and equipment's (cutting table and knife) swab samples from butcher shops were collected aseptically, processed, and analyzed bacteriologically. The hygiene and sanitation practiced was assessed using a structured questionnaire that was administered to workers in abattoir and butcher shops. Animals originated from the surrounding and different parts of the country such as Harar, Adama, Borana, Arsi, Bale, and others to be slaughtered. The study was conducted from November 2015 to November 2016 in Bishoftu municipal abattoir and butcher shops.

### 2.3. Questionnaire Survey

A structured questionnaire was prepared to assess the knowledge of workers in abattoir and butcher shops regarding the hygienic and sanitary practices during slaughter and processing of meat. The respondents were posed with the following questions to be answered. Educational status, exposure and frequency of training, effectiveness of training, practices of reporting illness and presence of hygienic regulatory system, if they use protective clothes, possess jewelry materials, money handling practices, and application of cleaning butcher shops. Simultaneously, observational study of the municipal abattoir and butcher houses was undertaken during the study period.

### 2.4. Sample Collection

A total of 124 samples were collected aseptically from abattoir and butcher houses. Sterile cotton tipped swabs soaked into buffered peptone water were used for swabbing in a template of 5 cm × 10 cm area of carcasses, cutting table, and knife as described in [14, 15]. The samples were properly labeled, kept in icebox, and transported to the National Veterinary Institute (NVI), Bishoftu, for bacteriological analysis.

### 2.5. Enumeration of Total Aerobic Plate Count

Each swab sample was added to 9 ml of sterile buffered peptone water under aseptic condition and well mixed with a vortex mixer [14]. Tenfold serial dilution up to 10^−8^ was made from 1 ml of the sample (original suspension) and 9 ml of buffered peptone water. From appropriate dilutions, 0.1 ml of the suspension was inoculated into labeled sterilized petridish in duplicate plates and 20 ml of melted plate count agar at (45–50)°C was poured on for each plate and mixed by rotating [16]. The plates were incubated at 37°C for 24–48 hours after the agar was solidified [17]. Four samples of carcass washing water were collected directly during washing carcass and analyzed for bacterial load in a similar way for the carcass swab samples except 1 ml of the suspension was plated from 10^−2^ and 10^−3^ in duplication [18]. The number of distinct colonies on each plate was enumerated using a colony counter, colonies ranged from 30–300 on each plate were accepted [19, 20], and colony forming units (cfu) per ml for water sample and per cm^2^ for the rest samples were calculated using the formula described in [16]. The results were converted to log_10_ cfu/cm^2^, and mean values of total aerobic plate counts were determined. The results were classified as below average and above average comparing with the standards described in [2], i.e., maximum limit of bacterial load that is acceptable with aerobic plate count of 5.0 log_10_ cfu/cm^2^ from raw meat.

### 2.6. Isolation and Identification of Bacteria

Bacterial isolation was performed using nutrient agar (HiMedia, India) and Tryptic soya agar (DIFCO, England) as general and enriched media. MacConkey agar (Sigma-Aldrich, United States) was used as a differential media. Selective media such as Baird–Parker agar (OXOID, England) for *Staphylococcus* species; Eosin methylene blue agar (HiMedia, India) for *Escherichia coli*, and Salmonella-Shigella agar (Titan Biotech, India) for *Salmonellae* and *Shigella* species were used for isolation and identification purpose. Presence of *Salmonellae* in the sample was established by preenrichment of the sample in lactose broth, followed by selective enrichment in tetrathionate broth and then cultured media on Brilliant green agar (OXOID, England). All the media used in the present study were prepared according to the manufacturer's specification, and collected samples were inoculated into plates and incubated at 37°C for 24–48 hours [19, 21]. Colonies identified as discrete on nutrient agar or Tryptic soya agar were carefully examined macroscopically (using stereo microscope) for cultural characteristics such as the shape, color, size, and consistency. Gram staining as well as appropriate biochemical tests was carried out according to the standard procedure [22]. The isolates were identified by comparing their morphological and biochemical characteristics with standard reference organisms of known taxa, as described in Bergey's Manual for Determinative Bacteriology [23].

### 2.7. Data Analysis

A database was developed to store qualitative and quantitative data from the cross-sectional study using Microsoft Excel 2010 spread sheet. STATA version 11 was used to compute descriptive statistics of variables collected during the study. Overall bacterial load was calculated using descriptive statistics of the sample through frequencies and cross tabulations.

## 3. Results

### 3.1. Questionnaire Survey

The abattoir workers were interviewed concerning their educational status, whether received training, presence of health certificate, reporting illness, protective clothing used, etc., as described in Table 1. Out of 31 interviewed abattoir workers, 48.4% of them did not received training. Forty-two percent of the workers had no health certificate, 64.5% of respondents report that they wore jewelry materials during working hours, and the abattoir has no organized written regulation system that can enforce the workers to keep the discipline of the work regarding hygiene. During the study, it was observed that there was no clear division of dirty and cleaning area of slaughtering process: stunning, bleeding, skinning, evisceration, or hanging, and cutting/deboning. Moreover, there was no cooling and sterilizing facility and preventive mechanism installed for insects and rodents in the municipal abattoir.

Table 2 summarizes various aspects of butcher shop workers regarding hygiene and sanitation conducted in their shops. A majority (66.67%) of the respondents were grade 1–4 (elementary) in their educational level and 58.33% of the respondents did not receive training regarding meat handling practices. 66.67% of butcher shop workers did not cover their hair using hairnet and handled money with bare hands during serving the meat to the consumers.

### 3.2. Aerobic Plate Count

Forty-eight swab samples from abattoir carcass, thirty-six swab samples from cutting knife, and thirty-six swab samples from cutting table were taken and analyzed as indicated in Tables 3 and 4. The total mean bacterial count log_10_ cfu/cm^2^ was found to be 4.53 and 6.37 from municipal abattoir and butcher house swab samples, respectively. The total aerobic plate counts (cfu/ml) from water samples were 2.1 × 10^2^ in round 1, 1.8 × 10^2^ in round 2, 3.2 × 10^2^ in round 3, and 2.6 × 10^2^ in round 4, and the total mean aerobic plate count of the water sample accounted 2.4 log_10_ cfu/ml.

**Figure 1:**
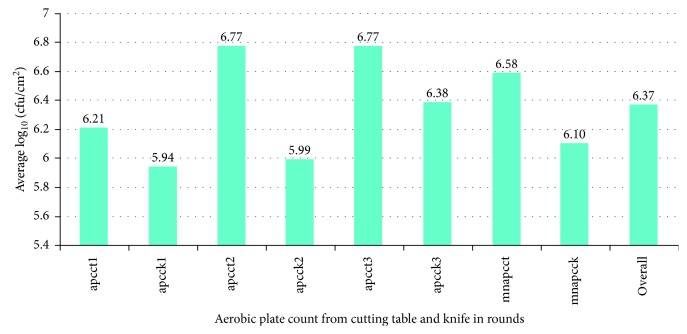
Aerobic plate count from cutting table and knife. ^*∗*^apcct = aerobic plate count from cutting table; apcck = aerobic plate count from cutting knife; mn = mean.

The mean bacterial count (log_10_ cfu/cm^2^) was found to be 6.1 and 6.58 for the samples from cutting knife and cutting table, respectively, as indicated in Figure 1.

**Figure 2:**
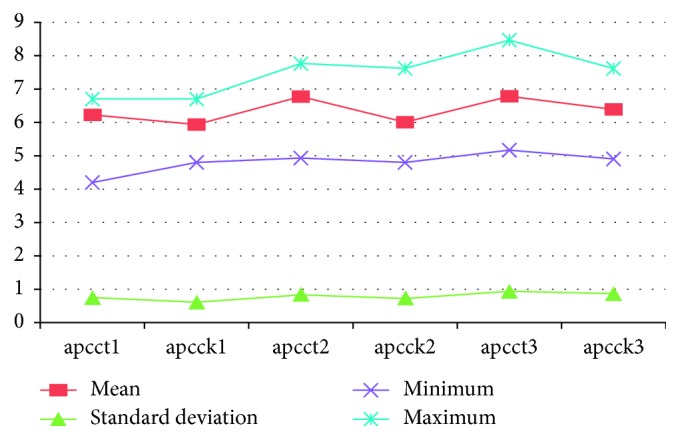
Characteristics of the bacterial load distribution described in mean and standard deviation.

The aerobic plate count showed with 4.2 and 8.45 log_10_ cfu/cm^2^ minimum and maximum bacterial load, respectively. The standard deviation and overall aerobic plate count from butcher shops are described as in Figure 2.

### 3.3. Bacterial Isolation

Bacterial contaminants found in the meat samples were *S. aureus*, *E. coli*, and *Salmonella* species. *E. coli* was the dominant isolate (35.2%), followed by *S. aureus* (22.5%). *Klebsiella* species, *Proteus* species, and *Shigella* species were also identified concurrently during the isolation and identification as described in Table 5.

## 4. Discussion

Abattoir is one of the food industries that contribute to the problem of possible food-borne diseases and health hazards associated with food unless the principles of food-borne hygiene practices are implemented [24]. The current study showed that there was no clear division of slaughtering process: stunning, bleeding, skinning, evisceration, hanging, and cutting/deboning. Furthermore, there was no preventive mechanism installed for insects and rodents in municipal abattoir which is similar with report in [25].

The hygienic condition of the abattoir workers has potential to contribute for contamination in meat processing. The author [26] reports unclean slaughter men's hands, clothing, and equipment used in carcass dressing process accounted for the microbial contamination. The study shows that 48.4% of abattoir workers did not cover their hair, 29% did not use apron, and 64.5% worn jewelry (ring, bracelets, watch, etc.) during working time. This finding is in agreement with [25] where 61.6% of abattoir workers did not cover their hair and wearing jewelry was not controlled at all.

The practice of wearing protective clothes helps to reduce the burden of contaminants in meat. Regarding this, the Ethiopian Ministry of Agriculture [27] recommends that personal clothing can carry microorganisms (germs) that have been gathered from a wide variety of sources into the meat or meat handling facility. Therefore, to protect meat and meat handling facilities from contamination because of personal clothing, protective overalls or hair cover should be worn at all times when handling meat. The overalls should be light in color so that contamination can be easily identified and the overalls cleaned easily. The wearing of jewelry, watches, and other detachable items should be discouraged. Dirt and organisms such as *S. aureus* can build up and around such items, and they pose a risk of foreign body contamination if they fall into the meat.

In addition to their clothes, the workers by themselves can be a probable source of contamination due to illness. It was recommended that new applicants could be examined clinically and bacteriologically before they are employed and at regular intervals afterwards. The examination should include medical history to determine past infections with special reference to dysentery, typhoid, and paratyphoid fevers, venereal and skin diseases, and bacteriological examination of stool and urine [28]. Most of the respondents agree in this study that even though the new applicants were asked for health certification, no periodic health status checkup was carried out in the abattoir. Out of those workers who reported illness (74.2%), 34.8% did not report through legal way (approved by medical examination). According to [28], emphasis should be placed that the workers with any sign of illness (diarrhea, vomiting, discharging wounds, sores etc.) should refrain from work until they are known not to be harboring dangerous pathogens.

Hygiene problems are not limited to slaughtering house but also associated with incorrect processing and marketing practices. According to the results of this study, 66.67% of the butcher shop workers handle money while serving food. Paper currency is widely exchanged for goods and services in countries worldwide. It is used for every type of commerce. All these trades are in hard currency, with lower denomination notes receiving the most handling because they are exchanged many times, and this makes it last less than a few years in circulation and provides a large surface area as a breeding ground for pathogens [29]. Handling of carcasses and money with the same unwashed hands could be good sources of contamination [30]. According to Muinde and Kuria's [31] report, handling of foods with bare hands may also result in cross contamination. Because meat handlers are probable sources of contamination for microorganisms, it is important that all possible measures should be taken to reduce or eliminate such contamination, which is supported by this study.

Similar to the abattoir, protective cloth is important in the butcher shops to reduce the chance of contamination. In order to protect both food products and meat handlers from cross contamination, the abattoir and butcher shop workers should wear protective clothes while working [32]. In this study, 66.67% of butcher shop workers did not cover their hair, which is in line with study conducted in Mekelle by Endale and Hailay [33]. Even though 83.33% of them had protective clothes (white coat), most of the workers in butcher shops had no habits of wearing it which is similarly reported in [34] from Tanzania.

Regular cleaning and disinfection of the beef retail outlets is important since it helps to reduce microbial contamination. Observation showed that most of the butcher shops are found on the road margin, exposed to dust due to wind or vehicle, and the organism found in it can contaminate them. Most of the surveyed butcher shops had poor hygienic condition concerning cleaning of their shops. This study is in agreement with [7] who reported lack knowledge of disinfection and sanitization by the butcher men.

Training of food handlers concerning basic concepts and requirements of personal hygiene plays a key role for ensuring safe food [35]. The level of education and training of food handlers about the basic concept and requirements of personal hygiene and its environment plays an important part in safeguarding the safety of products to consumers. The present study revealed that most of the abattoir and butcher shop workers had a low level of education. This could make difficult in acceptability of modern slaughtering practices as well as adherence to strict hygienic and standard slaughtering practices that contribute to microbial contamination, which is in line with report in [34].

The aerobic plate count (APC) is used as an indicator of the level of bacteria in meat and is a useful tool in monitoring food safety. To prevent the occurrence of food-borne illnesses and possible meat spoilage, it is important to ensure that foods sold are safe, wholesome, and in good hygienic condition. In this study, 37.5% of the abattoir carcass swab samples were found exceeding the limit (10^5^ cfu/cm^2^ or 5.0 log_10_ cfu/cm^2^) of total plate count on meat set by the WHO [2]. If the bacterial count exceeds the above standard in fresh meat, then the meat is not acceptable and this indicates alarm signals on meat hygiene along meat chain from abattoir to butcher shops. The total mean value in the present study of abattoir carcass swab sample was 4.53 ± 0.74 log cfu/cm^2^. Similar value has been reported by [36] from Algeria and [25] Mekelle abattoir in Ethiopia, which had a mean value of 4.48 ± 0.63 log cfu/cm^2^ and 5.04 log cfu/cm^2^, respectively. However, the result of the present study is lower than 5.80 ± 0.17 log cfu/cm^2^ reported in [37] at Mumbai abattoir in India. The difference could be due to the hygienic condition performed by workers in the abattoir.

The water used in slaughterhouse can also contaminate the meat during washing. The water used for cleaning procedures and meat processing in the abattoir must meet drinking water standards [38]. For this reason, an adequate supply of potable water should be available to meet operational and cleanup needs and it should be analyzed frequently to confirm its quality [39]. The total mean value of examined water samples during the study was 2.4 log_10_ cfu/ml. The present finding is higher than the report of Tarwate et al. [40], who reported a mean value of 2.1 log_10_ cfu/ml from water in abattoir. However, it is lower than the report of Pius [34] who had reported a mean value of 4.3 log_10_ cfu/ml in Ibadan, Nigeria. The variability of the results could be due to the quality of water used in the abattoir for washing carcass and regular monitoring and follow-up.

The high microbial load on the knife and cutting table is an indication of inadequate cleaning. Usually in the study area, knives are washed only with water and there is poor sterilization and continuous use of a single knife despite contact with dirty or contaminated surfaces. The presence of bacterial pathogens in meat contact surfaces may contribute to the contamination of meat [33]. In a similar way, the present study revealed the total mean of APC from butcher house equipment (cutting knife and cutting table). This result is similar with the value obtained from knife in [34] of 6.16 ± 1.25 log_10_ cfu/cm^2^ in Tanzania. The result of the cutting table is in agreement with findings in [33]. However, the highest bacterial load, 8.5 log_10_ cfu/cm^2^, from the cutting table of butcher shop was reported from the study conducted in Pakistan [7]. The variations of bacterial load observed in different studies might be due to lack of good processing and handling practices and sanitary standard operating procedures of meat along the meat production chain [34].

Even though the aim of this study was to isolate *S. aureus*, *E. coli*, and *Salmonellae* species, *Klebsiella*, *Proteus*, and *Shigella* species were also identified concurrently. Similar bacterial contaminants have been reported by different workers on food, water, and environmental samples [33, 41–43]. Among isolated bacteria, *E. coli* was the predominant organism followed by *S. aureus* and *Salmonella* species with minimum load from objectively isolated and identified bacteria in this study. Similar result was also reported by other investigators [33, 41] where they isolate these bacteria from meat and other environmental samples. The higher rate of contamination of meat with these organisms is an indication of deplorable state of poor hygienic and sanitary practices employed right from the slaughtering house, transportation to butcher shops, and processing at the butcher shops [25].

## 5. Conclusion and Recommendations

The results obtained from this study showed that there was high microbial load in abattoir and butcher shops. The high microbial logarithmic mean values (aerobic plate counts) from the samples tested are an indication of poor meat quality, making it a potential source of food-borne infection caused by *E. coli*, *S. aureus*, and *Salmonella* species and food spoilage. This was due to many factors such as the low level of sophistications, poor hygienic and sanitation procedures conducted at the abattoir and butcher shops, lack of training, and low educational level of the workers. From these results, it can be figured out that contamination was present right from the abattoir to the butcher shops where the meat produced in the study site is contaminated before it gets into the hands of consumers. Therefore, it is important to create awareness about hygiene and sanitation of meat both in abattoir and butcher shop, and appropriate control method of the problems should be designed and implemented. Moreover, further investigation should be carried out to isolate and characterize the bacterial load of meat in different study areas.

## Figures and Tables

**Table 1:** Summary on the various hygienic status of the municipal abattoir.

Characteristics	Frequency	Percent
Educational status (*n*=31)		
Grade 1–4	13	42
Grade 5–8	9	29
Grade 9–12	5	16
Above grade 12	4	13
Received training (*n*=31)		
Yes	16	51.6
No	15	48.4
Frequency of training (*n*=16)		
Annually	6	37.5
Every other year	10	62.5
Effectiveness of training (*n*=16)		
Yes	5	31.25
No	11	68.75
Presence of health certificate (*n*=31)		
Yes	18	58
No	13	42
Protective clothing used (*n*=31)		
Overall and gumboot	31	100
Apron	22	71
Hair cover	16	51.6
Use of jewelry material (*n*=31)		
Yes	20	64.5
No	11	35.5
Report illness (*n*=31)		
Yes	23	74.2
No	8	25.8
Health management action (*n*=23)		
Medical examination and treatment	15	65.2
Traditional approach	8	34.8
Presence of sanitary regulation system (*n*=31)		
Yes	0	0
No	31	100

*n*=number  of  respondents.

**Table 2:** Summary of the butcher shops workers responses (*n*=12).

Variable	Frequency	Percent
Education status (grade)		
Grade 1–4	8	66.67
Grade 5–8	2	16.67
Grade 9–12	1	8.33
Above 12	1	8.33
Received training		
Yes	5	41.67
No	7	58.33
Effectiveness of training (*n*=5)		
Yes	3	60
No	2	40
Hair of the butcher		
Covered	4	33.33
Not covered	8	66.67
Apron/white coat		
Use	10	83.33
Not used	2	16.67
Handling money		
Butcher with bare hand	8	66.67
Cashier	4	33.33
Jewelry (ring, watch, bracelets)		
Worn	10	83.33
Not worn	2	16.67
Application of cleaning		
Water only	9	75
Water and soap, detergent, etc.	3	25
Frequency of cleaning		
Daily	5	41.67
Every other day (once in 48–72 hrs)	7	58.33

**Table 3:** Aerobic plate counts from abattoir in (log_10_ cfu/cm^2^).

Round of sample collection	No. of observations	Mean (log_10_ cfu/cm^2^)	Standard deviation (SD)	Minimum count (log_10_ cfu/cm^2^)	Maximum count (log_10_ cfu/cm^2^)
*R* ^*∗*^1	12	4.41	0.62	3.7	5.49
*R*2	12	4.69	0.98	3.5	6.25
*R*3	12	4.38	0.67	3.6	5.38
*R*4	12	4.64	0.68	3.56	5.46
Average	—	4.53	0.74	—	—

^*∗*^ *R* = round of sample collection.

**Table 4:** APC from cutting knife and cutting table of butcher shops in log_10_ cfu/cm^2^.

Code of butcher house	apcct1	apcck2	apcct2	apcck2	apcct3	apcck3
A	6.47	5.6	7.49	5.74	7.17	7.6
B	6.57	6.1	7.75	5.75	6.6	6.25
C	6.67	5.6	6.85	5.6	7.2	7.5
D	5.47	6.7	5.68	6.18	6.5	6.6
E	6.69	6.6	7.5	6.14	7.3	6.4
F	6.5	6.67	6.6	5.3	6.56	6.6
G	6.47	6.3	7.52	6.3	7.63	5.6
H	6.53	5.6	6.38	5.6	5.67	5.5
I	5.8	5.4	6.49	7.6	7.3	6.5
J	6.5	6.3	6.41	6.5	5.7	5.6
K	4.2	4.8	4.92	4.8	5.17	4.9
L	6.7	5.6	7.65	6.4	8.45	7.5

apcct = aerobic plate count from cutting table in three rounds (1, 2, and 3). apcck = aerobic plate count from cutting knife in three rounds (1, 2, and 3).

**Table 5:** Isolated bacteria from collected samples.

Isolated bacteria	Abattoir (%)	Cutting table (%)	Cutting knife (%)	Water (%)	Total (%)
*E. coli*	7 (28)	9 (36)	7 (28)	2 (8)	25 (35.2)
*S. aureus*	4 (25)	7 (43.75)	5 (31.25)	0	16 (22.5)
*Salmonella* spp.	1 (14.3)	4 (57.1)	2 (28.6)	0	7 (9.9)
*Klebsiella* spp.	2 (15.4)	3 (23)	6 (46.2)	2 (15.4)	13 (18.3)
*Proteus* spp.	1 (12.5)	3 (37.5)	3 (37.5)	1 (12.5)	8 (11.3)
*Shigella* spp.	0	2 (100)	0	0	2 (2.8)

## Data Availability

The datasets used during the current study are available from the corresponding author on reasonable request.
